# Evaluation of a protocol for vancomycin administration in critically patients with and without kidney dysfunction

**DOI:** 10.1186/s12871-015-0065-1

**Published:** 2015-06-27

**Authors:** Savino Spadaro, Angela Berselli, Alberto Fogagnolo, Maurizia Capuzzo, Riccardo Ragazzi, Elisabetta Marangoni, Sara Bertacchini, Carlo Alberto Volta

**Affiliations:** Department of Morphology, Surgery and Experimental Medicine, Section of Anesthesia and Intensive Care, Sant’Anna Hospital, University of Ferrara, Ferrara, Italy

**Keywords:** Vancomycin, MRSA, Nephrotoxicity, Renal adjustment dose, Augmented renal clearance

## Abstract

**Background:**

Administration of vancomycin in critically ill patients needs close regulation. While subtherapeutical vancomycin serum concentration (VSC) is associated with increased mortality, accumulation is responsible for nephrotoxicity. Our study aimed to estimate the efficacy of a vancomycin-dosing protocol in reaching appropriate serum concentration in patients with and without kidney dysfunction.

**Methods:**

This was a retrospective study in critically ill patients treated with continuous infusion of vancomycin. Patients with creatinine clearance >50 ml/min (Group A) were compared to those with creatinine clearance ≤50 ml/min (Group B).

**Results:**

348 patients were enrolled (210 in Group A, 138 in Group B). At first determination, patients with kidney dysfunction (Group B) had a statistically higher percentage of vancomycin in target range, while the percentage of patients with a VSC under the range was almost equal. These percentages differed at the subsequent measurements. The number of patients with low vancomycin concentration progressively decreased, except in those with augmented renal clearance; the percentage of patients with VSC over 30 mg/L was about 28 %, irrespective of the presence or absence of kidney dysfunction. Patients who reached a subtherapeutic level at the first VSC measurement had a significant correlation with in-hospital mortality.

**Conclusions:**

Our protocol seems to allow a rapid achievement of a target VSC particularly in patients with kidney dysfunction. In order to avoid subtherapeutical VSC, our algorithm should be implemented by the estimation of the presence of an augmented renal clearance.

## Background

Patients of Intensive Care Units (ICU) are often characterized by a state of immunosuppression induced by underlying disease, therapies, and impairment of mechanical and immunological protective barriers, which exposes them to higher risk of hospital-acquired infections. Over the years, the repeated administration of antibiotic therapy has favoured the diffusion of Gram-positive Methicillin-Resistant Staphylococcus Aureus (MRSA), calling for stronger antibiotic therapies such as vancomycin, which is currently considered the treatment of choice for most MRSA infections [1].

Vancomycin, which is a poorly metabolized glycopeptide, is mainly excreted unchanged through urine and has its total body clearance correlated with glomerular filtration rate [2]. Indeed, creatinine clearance (CrCl) is frequently used as the clinical surrogate for glomerular filtration and its measurement can be used in clinical routines to help dose kidney-excreted drugs, such as vancomycin [3]. Accumulation of vancomycin may have major adverse effects such as nephrotoxicity and ototoxicity, in both patients with and without renal dysfunction [4]. The incidence of nephrotoxicity can vary between 5 % and 35 % both in patients with and without kidney dysfunction [5]; the adverse effects of nephrotoxicity may include an increase of creatinine, azotemia as well as changes in urinary sediment (hematuria, proteinuria and casts). The incidence of ototoxicity ranges between 1 % and 9 % in both patients population [5].

However, more recently, it has been underlined that antibiotics overdosing could be of less clinical relevance when compared to underdosing, since the latter is associated with an increased mortality [6–9]. Recent studies suggest that alterations of fluid distribution, hemodynamic parameters, microcirculation and organ functions may be associated with subtherapeutic level of vancomycin [4, 8, 10]. The latter is responsible of treatment failure, selection of resistant organisms and higher mortality [1, 10–12], underlying that low plasmatic levels of vancomycin are even more relevant for patient outcome than increased plasmatic concentration [13]. Furthermore, subtherapeutic levels of vancomycin has been associated with augmented renal clearance (ARC) [10], which is characterised by an enhanced renal elimination of circulating solutes observed in critically ill patients [6, 9, 12].

Hence, when treating an ICU patient with vancomycin, physicians should balance accumulation with subtherapeutic level of vancomycin; though this can be particularly challenging in patients with kidney dysfunction, as vancomycin clearance is mainly related to kidney function and such patients are at a higher risk of nephrotoxicity (compared to those with normal kidney function) [14, 15].

Accordingly, we developed a protocol for vancomycin infusion that takes into account the clearance of creatinine for determining the daily dose of vancomycin, along with vancomycin serum concentration (VSC) measurements for the subsequent dose adjustments needed to overcome the risks of ineffectiveness (failure of treatment) or nephrotoxicity.

The aims of the present study were: (1) to estimate the efficacy of the vancomycin dosing protocol to reach a correct serum therapeutic range in critically ill patients with and without kidney dysfunction (2) to analyse which factor(s) are related to subtherapeutic VSC.

## Methods

This retrospective observational cohort study was performed on clinical records of patients who had been admitted between March 2010 and July 2013 to a 6-bed mixed ICU, part of a 740-bed University hospital. The study was approved by the local Ethics Committee of Ferrara who waived the need for informed consent in consideration of the retrospective and observational nature of the study.

Inclusion criteria were patients having received parental vancomycin for an empiric or target sepsis treatment for over 48 hours; whereas exclusion criteria were: having been treated by Continuous Renal Replacement Technique, being under 18 years of age, and pregnancy. Clinical records of evaluated patients were reviewed to collect information concerning gender, age, weight, height, SAPS II scores, characteristics of infection, serial serum creatinine levels and vancomycin dose administered by infusion in the 24 h before measurement. CrCl was obtained daily from 24 h urine collection as a normal procedure in our ICU and normalised to body surface area, at the beginning and during vancomycin therapy [10, 13, 16]. The equation for calculating the CrCl uses the urinary volume, the urinary creatinine concentration and the serum creatinine concentration. The presence of an Augmented Renal Clearance was defined as a CrCl of more than 130 mL/min per 1.73 m^2^ [10, 12, 13].

**Fig. 1:**
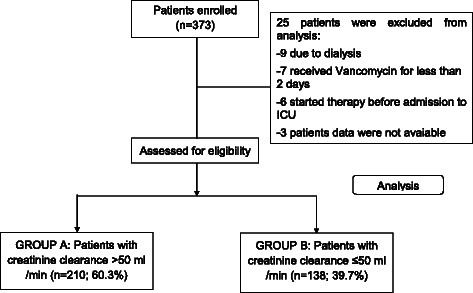
Flowchart of the study

In accordance with Maki et al [16], patients were divided in two groups: those with a creatinine clearance >50 ml/min (Group A), and those with a creatinine clearance ≤50 ml/min (Group B) [16]. The flowchart of the study is illustrated in Fig. 1. Data was coded and downloaded as an electronic file for further analysis. The presence of concomitant exposure to potential nephrotoxic drugs (including angiotensin converting enzyme inhibitors, aminoglycosides, vasopressor) were also collected [4].

In accordance with data in literature, the routine ICU vancomycin dosing protocol (Table 1) foresaw administration by continuous infusion, which is more compatible with the antibiotic’s pharmacokinetic characteristics. Throughout the period considered by our study, the protocol set a weight-based loading dose of 1000 mg for patients with a body weight <65 Kg, and 1500 mg for patients with a body weight >65 Kg [5], diluted in a 100 ml of saline solution and administered over 60 minutes when the loading dose was 1000 mg; and over 90 min when the loading dose was 1500 mg. The loading dose was followed by the infusion of a dose of vancomycin calculated according to the CrCl [17]: specifically, 2000 mg/day if creatinine clearance was >50 ml/min/1,73 m^2^, 1500 mg/day if creatinine clearance was between 20–50 ml/min/1,73 m^2^, 1000 mg/day if creatinine clearance was between 10–20 ml/min/1,73 m^2^ and 500 mg/day if creatinine clearance was < 10 ml/min/1,73 m^2^. Consecutive infusion rate was adjusted according to VSC: the daily dose was increased by 500 mg when VSC was <15 mg/L, left unchanged when VSC was between 15–25 mg/L, and was decreased by 500 mg when VSC was >25 mg/L and the infusion was interrupted for 6 hours when VSC exceeded 30 mg/L [5, 7] (Table 1).

**Table 1:** Protocol for Vancomycin administration in use in our department

CLINICAL PROTOCOL	
STARTING LOADING DOSE:	
if body weight < 65 Kg = 1000 mg	
if body weight ≥ 65 Kg = 1500 mg	
DAILY DOSE:	
Creatinine clearance	Vancomycin daily dose
>50 ml/min/1,73 m^2^	2000 mg
50-20 ml/min/1,73 m^2^	1500 mg
20-10 ml/min/1,73 m^2^	1000 mg
<10 ml/min/1,73 m^2^	500 mg
DOSE ADJUSTMENT AFTER VANCOMYCIN SERUM CONCENTRATION (VSC) MEASUREMENT:	
VSC	Adjustment dose
<15 mg/L	Increase infusion 500 mg/day
15-25 mg/L	Do not change infusion
25-30 mg/L	Riduce infusion 500 g/day
>30 mg/L	Stop infusion for 6 h, than riduced dose

According to our protocol, blood samples are collected from the arterial line. Moreover, all the blood samples for VSC measurements and creatinine were drawn at 7:30 in the morning in all patients receiving vancomycin continuous infusion. The first sample of vancomycin serum concentration of each patient was collected on the second day after starting the infusion [10], as suggested by Kitsiz et al [18] who demonstrated that the first VSC after the LD should be performed within 36–48 hours. As for our ICU protocol, the subsequent samples were collected every 48 hours. VSC was measured using Dimension® clinical chemistry system (Dade Behring, Ramsey MN, USA) with methodology based on immunoassay technique (PETINIA). The VSC laboratory reports were made available in electronic format to the ICU attending physician in the afternoon of the same day of blood sample collection. We considered 15–25 mg/L as the target range of VSC, based on the assumption that a steady-state 24-h area under the concentration-time curve divided by the minimum inhibitory concentration ratio (AUC_24h_/MIC) of ≥400 is associated with increased clinical success [7]. Vancomycin concentrations were then considered as “insufficient” at <15 mg/L, “adequate” at 15–25 mg/L, slightly increased at 25–30 mg/L and “excessive” at >30 mg/L [5, 19]. The safety of the protocol was evaluated in terms of nephrotoxicity. The latter was defined as an increase of 0.5 mg/dl or 50 % increase from baseline in serum creatinine for two consecutive assays, as proposed in a recent Consensus Review [5].

### Statistical analysis

Statistical analysis was performed by using SPSS 18.0 for Windows NT (SPSS IncChicago.IL 2004 USA). Because our data were normally distributed, as evidenced by the Kolmogorov-Smirnov test, data are presented as means and standard deviation (SD) or percentage. The statistical analysis was conducted using Chi-square tests or Fisher’s Exact tests for categorical variables. Multivariate logistic regression analysis was conducted to assess variables associated with occurrence of nephrotoxicity. Variables evaluated in multivariate analysis were vasopressor (norepinephrine, phenylephrine, or dopamine) infusion, aminoglycosides, angiotensin converting enzyme inhibitors (ACEi), serum vancomycin and creatinine clearance at ICU admission. Multivariate logistic regression analysis was also conducted to assess variables associated with in-hospital mortality. A p value of <0.05 was considered to show a statistically significant difference.

## Results

The study considered a total of 348 patients. Two hundred and ten had a creatinine clearance >50 ml/min (Group A) and 138 had a creatinine clearance ≤50 ml/min (Group B). Demographic and clinical characteristics of patients are summarized in Table 2.

**Table 2:** Demographic data, patient clinical characteristics, site of infection and microorganisms identified

	GROUP A	GROUP B
N. of patients	210	138
Gender male. n. (%)	153 (73)	95 (69)
Age (year)	63.0 ± 11.2	70.6 ± 9.9*
BMI (Kg/m^2^)	27.2 ± 6.3	26.0 ± 5.6
Basal Serum creatinine (mg/L)	1.0 ± 0.4	2.3 ± 1.4*
Basal Creatinine Clearance (ml/min/1.73 m^2^)	106.4 ± 40.7	37 ± 16.2*
Loading dose (mg/kg)	16.7 ± 4.8	18 ± 2
Mean time to first VSC (hours)	40 ± 3	41 ± 2
Total continuous infusion dose^a^ (g)	5.4 ± 0.3	3.6 ± 0.8*
Duration of vancomycin therapy (days)	9.0 ± 1.1	9.1 ± 1.6
Nephrotoxicity n. (%)	5 (2.4)	13 (9.4)
Concomitant use of nephrotoxic agents:		
Vasopressor n. (%)	32 (15.2)	25 (18.1)
Aminoglycoside. n. (%)	12 (5.7)	9 (6.5)
Ace inhibitor n. (%)	41 (19.5)	21 (15.2)
Type of patient at ICU admission:		
Medical n. (%)	119 (56.7)	69 (50.0)
Urgent surgery n. ( %)	7 (3.3)	23 (16.6)^*^
Scheduled surgery n.(%)	84 (40.0)	46 (33.4)
Admission diagnosis. n. (%)		
Sepsis	63 (30 %)	36 (26)
Respiratory failure without sepsis	43 (20.4)	26 (18.8)
Post surgery	80 (38)	66 (47.8)
Other	24 (11.4)	10 (7.2)
SAPS II (first 24 h)	40.3 ± 8.0	46.2 ± 7.7*
ICU deaths. n. (%)	45 (21.4)	33 (23.9)
Site of infection. n.( %)		
Low Respiratory Tract	102 (48.6)	50 (36.2)*
Intra-abdominal	43 (20.5)	44 (31.8)*
Bloodstream	30 (14.3)	6 (4.3)*
Skin and soft tissue	23 (10.9)	38 (27.5)*
Other	12 (5.7)	0*
Microorganisms identified. n. (%)		
*Staphylococci* spp	133 (63.3)	86 (62.3)
*Enterococci* spp	74 (35.2)	52 (37.7)
*Streptococci* spp	3 ( 1.4)	0
Vancomycin AUC/ MIC at first determination	468 ± 79	490 ± 84

Continuous variables are reported as mean ± standard deviation (SD); * p<0.05

*BMI*: Body Mass Index

^a^ Total infusion of vancomycin dose until first determination (included loading dose)

**Fig. 2:**
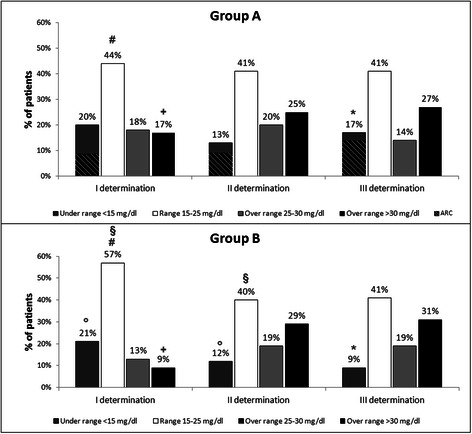
Distibution of vancomycin concentration in patients with and without nephrotoxicity. All Statistically significant *p*-value (<0.05) are marked in the figure. # p = 0,026; + p = 0,005; § = 0,024; o p < 0,001; *p = 0,005

At the first determination, patients with kidney injury (Group B) had a statistically higher percentage of VSC in the target range (Fig. 2), while the percentage of patients with a VSC under the range was mostly the same. In this connection it should be pointed out that almost half of the patients in Group A had an ARC (Fig. 2).

Such percentages differed at the second and third measurements of VSC. Both groups reduced the number of patients with a VSC lower than the adequate range, while the percentage of patients with VSC over 30 mg/L was about 28 % (Fig. 2), independently from the presence or absence of kidney dysfunction. The number of patients with low VSC progressively decreased, except for those with ARC. Indeed, at the second determination, 66 % of the patients with VSC below the desired range had an ARC, while this percentage increased up to 80 % at the third determination. The duration of vancomycin therapy was about 9 days for both groups (Table 2). Of note, the % of patients with a VSC higher than 35 mg/dl was extremely low in both groups (Group A: 0.95 % at the first determination, 1.42 % and 0.95 % at the second and third determination, respectively. Group B: 0.72 % at the first determination, 1.44 % at the second and third determination).

Although the rate of nephrotoxicity was 2,4 % and 9,4 % in Group A and B respectively, no correlations were found between nephrotoxicity and VSC within the target range and over the range (p = 0.65). Furthermore less than 8 % of patients experiencing a VSC >30 mg/L had a relevant variation of creatinine. Of note, no nephrotoxicity was observed in presence of a VSC ranging between 25 and 30 mg/l.

According to the multivariable logistic regression analysis, nephrotoxicity was not associated with any of the following variables: vasopressor infusion (p 0.9), aminoglicosides (p 0.9), angiotensin converting enzyme inhibitors ace (p 0.9) and serum vancomycin (p 0.7). The only variable related to nephrotoxicity was the creatinine clearance at ICU admission (p 0.04). Regarding the possible side effects of vancomycin administration, we report no ototoxicity in both groups. Finally, patients who reached a subtherapeutic level at the first VSC measurement had a significant correlation with in-hospital mortality (OR 2.1; p 0.003) (Table 3). In this connection it should be pointed out that the AUC/MIC in both groups (group A: 468 ± 79 and group B: 490 ± 84, respectively) was in the desired range (>400) [5] at the first VSC determination (Table 2).

**Table 3:** Variable associated with in hospital mortality (multivariate analysis)

Variables	Odds Ratio	95 % CI	*p* value
VSC under range at first determination	2.147	1.953 – 2.350	0.003^a^
SAPS II	1.038	1.019 – 1.058	0.003^a^
Age	1.017	997 – 1.037	0.396
BMI	0.957	0.913 – 1.003	0.08
Creatinine clearance	0.990	0.984 - 0.996	0.832

^a^Significance with p-value < 0.05

## Discussion

The main results of the present study are: 1) our protocol seems to consent to a fast achievement of a target VSC in patients with normal kidney function and in those with kidney dysfunction; 2) the presence of an augmented renal clearance was the main determinant of the difficulties in reaching a target VSC.

Early antibiotic administration should aim to reach adequate target VSC within a few hours from infusion [10, 17, 19]. However, critically ill patients usually have a large volume of distribution which likely reflects significant capillary leakage. The latter, coupled with aggressive fluid loading, can expand the interstitial space [4, 20]. Hence hydrophilic antimicrobials — distributed exclusively in the extracellular compartment— are expected to be diluted. This shift of fluid may favour movement of drug into the interstitium and a decreased VSC is expected. Moreover, while critical illness might alter the volume of distribution, renal dysfunction additionally makes antibiotic pharmacokinetics even more unpredictable [8]. As a consequence, patients with decreased renal function require vancomycin to be administered following dose adjustments. Hence our protocol was designed in order to avoid both a VSC under and over the target range, although we choose to favour the avoidance of a VSC under the range since its association with an increased in-hospital mortality. At the same time we felt that patients with kidney dysfunction should have had a specific protocol because of reduced renal clearance. Finally, target VSC may be difficult to achieve because of the presence of augmented renal clearance [9, 12, 21]. ARC is characterised by an enhanced renal elimination of circulating solutes observed in critically ill patients [8, 22]. The presence of ARC might imply subtherapeutic levels of a given drug for substantial periods of the dosing interval resulting in treatment failure or selection of resistant organisms [1, 12, 22].

All the above mentioned factors imply great inter-individual variability in pharmacokinetics, complicating accurate prediction of serum concentrations in ICU patients, making evident the need of a frequent dosage of VSC in order to make the daily dose of this antibiotics adequate [5].

A below-range VSC was observed in only 20 % of patients of both groups. However, only patients with kidney dysfunction progressively and significantly reduced this percentage, which became about 9 % at the third VSC determination. Interestingly, the group with normal kidney function had a different behaviour. Between the first and the third VSC determination, it became progressively evident the relevance of an ARC that did not allow this group to decrease the percentage of patients with VSC under the range. This is of clinical relevance since patients who reached a subtherapeutic level at the first VSC measurement had a significant correlation with in-hospital mortality (OR 2.1; p 0.003).

As to the VSC in the desired range, only at the first determination group B had higher percentage of patients with VSC in the normal range and, as a consequence, a lower percentage with a VSC over the range (Fig. 2). At the second and the third VSC determination the two groups were almost identical. This was the case also for VSC over the target range, being the percentage of patients with a VSC higher than 30 mg/L of about 28 %. The increase of VSC over the target range has been associated with a risk of nephrotoxicity that has a reported incidence up to 35 % during vancomycin therapy [23, 24]. Hence we investigated the effects of VSC over the target range on renal function. Interestingly no correlation was found between VSC and renal toxicity, even in patients with kidney dysfunction. This is relevant from the clinical point of view, since other and more expensive antibiotics are usually proposed in patients at risk of kidney dysfunction. Such result can be explained by several factors. First of all, we considered 15–25 mg/L as a target range since a VSC over 25 mg/L can be associated with increase of nephrotoxicity, as previously described [6]. However, other studies have proposed an upper limit of 30 mg/L [12], suggesting that nephrotoxicity could be enhanced when the VSC reaches values higher than 30 mg/L, as it was the case of the present study. The VSC > 30 mg/L was found in about 30 % of the patients of both groups (Fig. 2), whilst the % of patients with a VSC higher than 35 mg/dl was extremely low in both groups (about 1 %, see results section). These results underline that our algorithm is able to adjust the VSC preventing the progressive accumulation of vancomycin without incurring the opposite phenomenon, that is a VSC below 15 mg/dl.

Our algorithm is based on a continuous infusion of vancomycin that might have advantages over intermittent administration [25, 26] since this strategy appears to reduce renal toxicity [25]. Despite previous studies comparing continuous and intermittent administration have reached conflicting results [27], others demonstrated that continuous infusion of vancomycin is less expensive and quicker in achieving target concentration, resulting in less variability in serum concentrations [27, 19].

The presence of patients with a VSC > 30 mg/L implies that our algorithm should not be changed by increasing the daily dose of vancomycin in order to avoid a VSC under the desired range. This is suggested by the AUC/MIC ratio, which was even at the first VSC determination much higher than 400 (Table 2). This is of clinical relevance since Holmes et al [28] have previously demonstrated a 12 % lower mortality 30-day mortality in patients achieving a vancomycin AUC/MIC of >373 within the first 96 h of vancomycin therapy compared to those who did not. Instead, we believe that our algorithm should take into account the presence of ARC, therefor increasing the dose only in patients that really need this adjustment of the therapy.

### Limitation of the study

We used a loading dose of about 15 ml/Kg of actual body weight to avoid too high VSC. However, the LD varies among different studies and other authors suggest a LD of about 25–30 mg/Kg (low level of evidence – III - and grade of recommendation B) [5], with the aim of rapid achievement of the target VSC. Looking at our data, between 40 and 50 % of patients of both groups were over-range, generating a clinical dilemma. Should we modify our algorithm by increasing the loading dose or should we maintain the LD used in the present study? Indeed, it can be hypothesized that higher LD would have determined higher plasmatic concentration. Hence it could be expected an increased % of patient in over-range or even of patients in over range with VSC much higher than those obtained in the present study, leading to an increase of nephrotoxicity. Moreover, the AUC/MIC was much higher than 400 for both groups Table 2), suggesting that the LD might have been sufficient to reach the expected VSC. Further studies are required to clarify if an increased LD could decrease the % of patients in under-range without increasing nephrotoxicity.

Finally, calculation of creatinine clearance implies determination of urinary creatinine. The latter, however is influenced by the volume status of the patient, treatment with the loops diuretics and vasopressor agents, and release of antidiuretic hormone. Indeed, correct estimation of glomerular filtration rates implies its determination by using inulin or iohexol clearance, and radionucleotide. Unfortunately, these methods are largely unavailable in the clinical setting.

## Conclusions

Our protocol seems to allow a rapid achievement of a target VSC particularly in patients with kidney dysfunction. In order to avoid subtherapeutic VSC, our algorithm should be implemented by the estimation of ARC, the presence of which implies an adjustment of the dose of vancomicyn, both in terms of loading and continuous infusion dose.

## Abbreviations

ARC: Augmented renal clearance
AUC: Area under the curve
CrCl: Creatinine clearance
ICU: Intensive care unit
MIC: Minimum inhibitory concentration
MRSA: Methicillin-resistant staphylococcus Aureus
VSC: Vancomycin serum concentration
